# Elabela-Apelin Receptor Signaling Pathway is Functional in Mammalian Systems

**DOI:** 10.1038/srep08170

**Published:** 2015-02-02

**Authors:** Zhi Wang, Daozhan Yu, Mengqiao Wang, Qilong Wang, Jennifer Kouznetsova, Rongze Yang, Kun Qian, Wenjun Wu, Alan Shuldiner, Carole Sztalryd, Minghui Zou, Wei Zheng, Da-Wei Gong

**Affiliations:** 1 Division of Endocrinology, Diabetes and Nutrition, Department of Medicine, University of Maryland School of Medicine, Baltimore, MD 21201; 2 Department of Cardiology, Nanjing Chest Hospital, Southeast University School of Medicine, Nanjing, 210029; 3 National Center for Advancing Translational Sciences, National Institutes of Health, Bethesda, MD 20892; 4 Department of Internal Medicine, University of Oklahoma Health Science Center, OK 73104

## Abstract

Elabela (ELA) or Toddler is a recently discovered hormone which is required for normal development of heart and vasculature through activation of apelin receptor (APJ), a G protein-coupled receptor (GPCR), in zebrafish. The present study explores whether the ELA-APJ signaling pathway is functional in the mammalian system. Using reverse-transcription PCR, we found that ELA is restrictedly expressed in human pluripotent stem cells and adult kidney whereas APJ is more widely expressed. We next studied ELA-APJ signaling pathway in reconstituted mammalian cell systems. Addition of ELA to HEK293 cells over-expressing GFP-AJP fusion protein resulted in rapid internalization of the fusion receptor. In Chinese hamster ovarian (CHO) cells over-expressing human APJ, ELA suppresses cAMP production with EC_50_ of 11.1 nM, stimulates ERK1/2 phosphorylation with EC_50_ of 14.3 nM and weakly induces intracellular calcium mobilization. Finally, we tested ELA biological function in human umbilical vascular endothelial cells and showed that ELA induces angiogenesis and relaxes mouse aortic blood vessel in a dose-dependent manner through a mechanism different from apelin. Collectively, we demonstrate that the ELA-AJP signaling pathways are functional in mammalian systems, indicating that ELA likely serves as a hormone regulating the circulation system in adulthood as well as in embryonic development.

Apelin receptor (APJ or APJ receptor) was initially cloned as an orphan G-protein coupled receptor which shares 30% homology to angiotensin receptor 1^1^. Its ligand, apelin, was isolated from bovine stomach extract through high-throughput screening assay in 1998^2^. Both APJ and apelin are expressed in many tissues including heart, lung, endothelium, kidney and brain. A decade of investigations have shown that the apelinergic system has a broad range of biological functions, playing an important role particularly in maintaining homeostasis of the cardiovascular system and fluid metabolism^3^,^4^. The activation of APJ causes a broad spectrum of biochemical changes including cAMP suppression^5^, phosphorylation of protein kinase B (Akt), ERK1/2^5^ and p70S6K^6^, calcium mobilization^7^ and nitric oxide synthase (NOS)^8^,^9^. However, the specificity linking these biochemical activities to a certain biological function is yet to be determined.

Apelin was thought to be the only ligand for APJ until recently when two research groups independently discovered, through mining of long non-coding RNA transcripts, that a short secretory peptide, called Elabela (ELA)^10^ or Toddler^11^ is another ligand for APJ (for convenience, we choose to use the ELA nomenclature in the present manuscript). Through elegant genetics studies using the zebrafish model system, both teams have demonstrated that ELA signaling is required for normal heart and vasculature development. ELA deficiency leads to severe defects in heart development and lymphogenesis. Remarkably, the ELA mutant phenotype is similar to the APJ mutants in zebrafish and therefore suggests that ELA and APJ are genetically in the same biological pathway. Indeed, ELA binds and activates APJ, establishing that ELA is the ligand of APJ required for normal cardiac development in zebrafish.

ELA is a peptide of 54 amino acids including a secretory signal with a mature form containing 32 amino acids^10^. The cDNAs encoding ELA are found and well conserved in vertebrates^10^,^11^, suggesting a functional conservation of the ELA-APJ pathway in mammalians. ELA transcripts are found in human pluripotent stem cells and adult kidney and prostate^10^, but whether human ELA acts on APJ receptor and is biologically functional in mammalian cells has not yet been determined. We report, here, an initial characterization of ELA-APJ signaling pathways and functions in mammalian systems. Our study shows that ELA activates APJ signaling pathways, induces angiogenesis of human HUVECs and relaxes mouse aortic blood vessel, thus establishing that ELA-APJ signaling pathways are conserved and functional in vertebrates.

## Results

### Tissue distribution of ELA and APJ in humans

**Figure 1:**
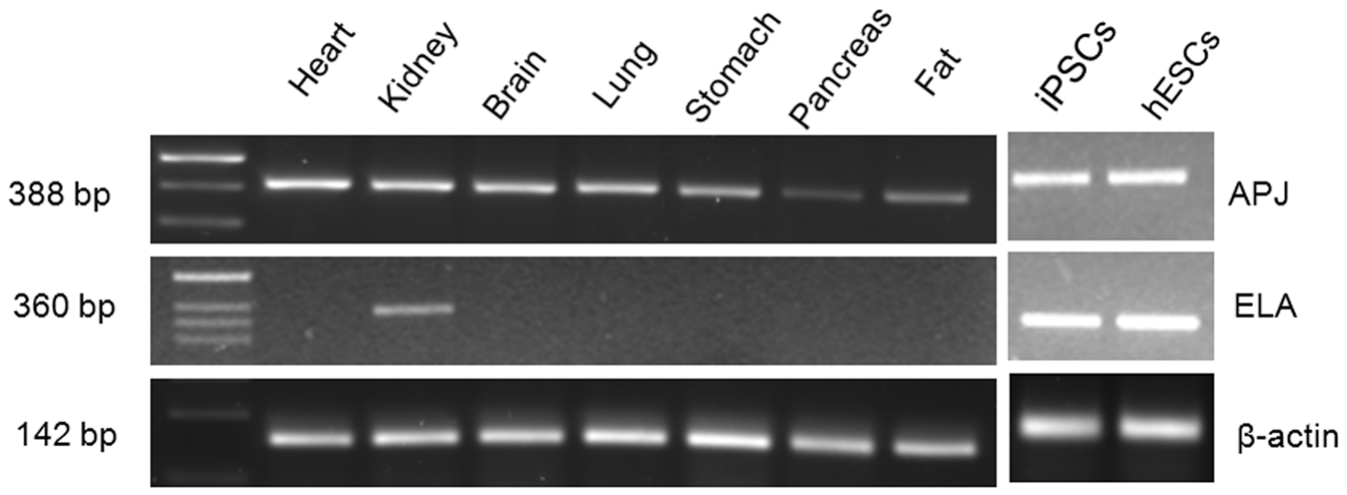
Tissue-restricted expression of ELA in the human. Reverse transcription PCR was conducted on total RNAs extracted from the indicated human tissues, and embryonic (ESCs) and induced (iPSCs) pluripotent stem cells using primers specific for human ELA, APJ and β-actin.

To determine the tissue distribution of ELA in humans, in comparison to APJ, we conducted RT-PCR analyses of ELA and APJ on cDNAs derived from multiple tissues with primers specific for ELA or APJ. As shown in Fig. 1, APJ is widely distributed and expressed in tissues of heart, brain, kidney, stomach, lung, adipose tissues and pancreas. In contrast, ELA is expressed only in the adult kidney tissue, and embryonic and induced adult pluripotent stem cells (ESCs and iPSCs).

### Induction of human APJ endocytosis by ELA

**Figure 2:**
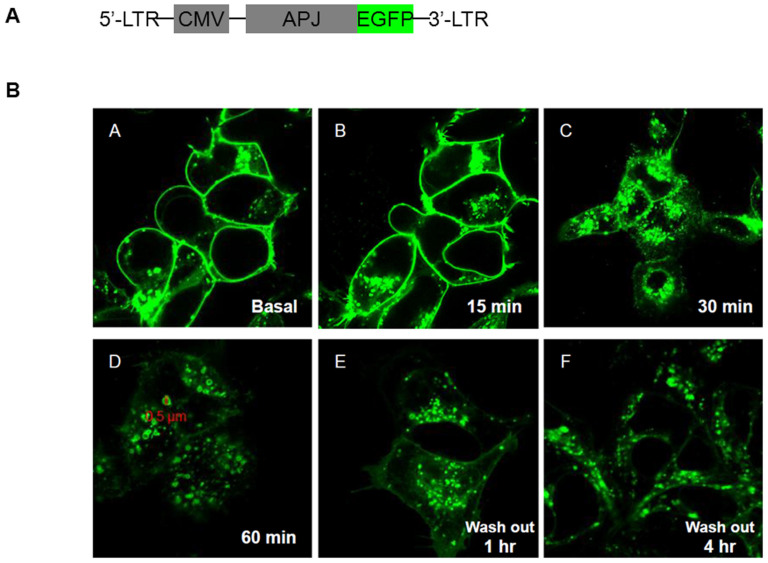
Internalization of APJ-EGFP fusion protein by ELA. (A). Scheme of lentiviral construct of APJ-EGFP construct. (B). Response of APJ-EGFP to ELA. HEK293 cells expressing APJ-EGFP were viewed live under confocal microscopy at indicated times of 0 (A), 15 min (B), 30 min (C), and 60 min (D) after addition of ELA (0.5 μΜ), and 1 hr (E) and 4 h (F) after washout of the cells which had been treated with ELA for 60 min. Scale bar indicates the internalized of receptor in larger, hollow vesicles of ~0.5 μm.

Ligand-induced receptor endocytosis is a typical cellular response of GPCR to ligand binding and activation. To investigate whether ELA will induce APJ internalization, we over-expressed APJ as a fusion protein with enhanced green fluorescent protein (APJ-GFP, Fig. 2A) through lentiviral infection in HEK293 cells and examined its intracellular localization in response to ELA treatment. At the basal level, the fusion protein was localized largely at the cell surface, with some perinuclear expression detected (Fig. 2B). Following ELA treatment, intracellular dot-like vesicles appeared by 15 min and became more apparent by 30 min. By 60 min, large and hollow intracellular vesicles (~3–5 μm in diameter) were clearly visible in the cytoplasm. The fluorescent vesicles remained in the cytoplasm 1 hr and appeared to start to return to the cell surface 4 hr after the washout in cells previously treated with ELA for 60 min.

### Suppression of cAMP production, activation of ERK1/2 and calcium mobilization by ELA

**Figure 3:**
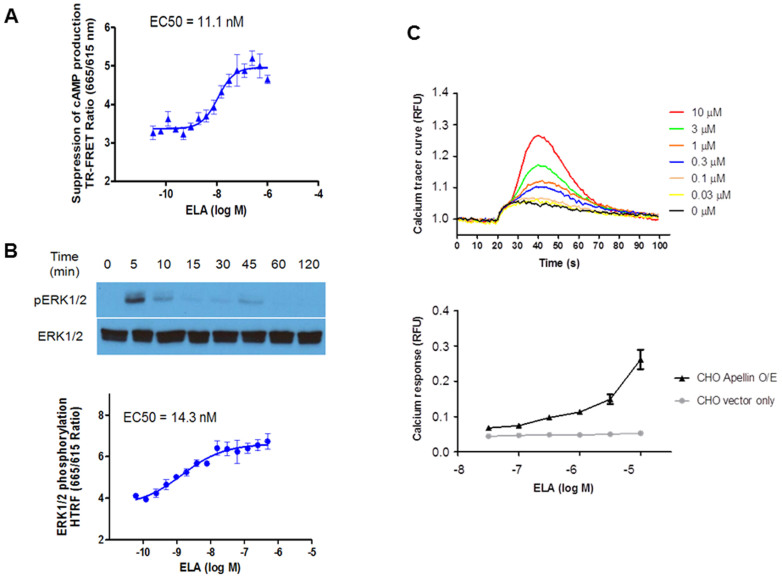
Down-stream signaling of APJ activation by ELA. (A). Suppression of cAMP production by ELA. CHO cells expressing APJ were treated with forskolin (10 μM) and ELA at the indicated concentrations. The amount of cAMP was measured by TR-FRET. Higher TR-FRET ratio indicates less cAMP production. (B). Phosphorylation of ERK1/2 by ELA. Upper panel: APJ-expressing CHO cells were treated with ELA (1 μΜ) at the indicated times and lysed for Western blot analysis by antibodies of phosphorylated form of ERK (pERK1/2) and unphosphorylated ERK1/2 (ERK1/2). (B). Dose response of ERK phosphorylation by ELA. APJ-expressing CHO cells were treated with ELA at the indicated concentrations and assayed for HTRF ratio by TR-HTRF. (C). Intracellular calcium mobilization by ELA. CHO cells expressing human APJ were treated with ELA at the indicated concentration and calcium mobilization was monitored by fluorescent calcium dye Fluo8 for up to 100 seconds (upper panel). Lower panel: calcium response to ELA in APJ-expressing cells vs. vector control cells. Values are mean ± SEM (n = 3–5).

Activation of APJ is known to result in a decrease of cAMP production, stimulation of ERK and increase in intracellular calcium mobilization^12^. We next investigated whether ELA could activate these signaling pathways. APJ-over-expressing and empty vector control CHO-K1 cells were used in the studies. APJ expression was confirmed by RT-PCR. For cAMP assays, the CHO-K1 cells were pre-treated with increasing concentrations of ELA and then with the adenylate cyclase agonist forskolin to assess the biological activity of ELA to suppress cAMP production. As shown in Fig. 3A, ELA inhibited cAMP production in a dose-dependent manner with an EC_50_ of 11.1 nM. In addition, we determined in the same cell system that ELA was able to induce the phosphorylation of ERK1/2 within 5 min of treatment, and this effect lasted for 45 min as revealed by Western analysis (Fig. 3B, upper panel). We confirmed quantitatively the effect of ELA on ERK1/2 phosphorylation by time-resolved fluorescence resonance energy transfer (TR-FRET) assay. Fig. 3B shows that ELA stimulated ERK1/2 in a dose-dependent manner at the tested concentration range from 10^−10^ to 10^−6^ M, with an EC_50_ of 14.3 nM. Finally, we investigated ELA effect on intracellular calcium mobilization. ELA treatment caused a rapid calcium mobilization, peaking at 40 seconds in a dose-dependent manner (Fig. 3C, upper panel). The response was dose-dependent up to the concentration of 10^−5^ M (Fig. 3C, lower panel). Most importantly, we did not observe meaningful changes in parallel control experiments in the above assays for cells transduced with empty vector control viruses and vehicle (Fig. 3C and data not shown). Thus, ELA's effects on intracellular signaling were APJ-dependent.

### Angiogenic effect of ELA *in vitro*

**Figure 4:**
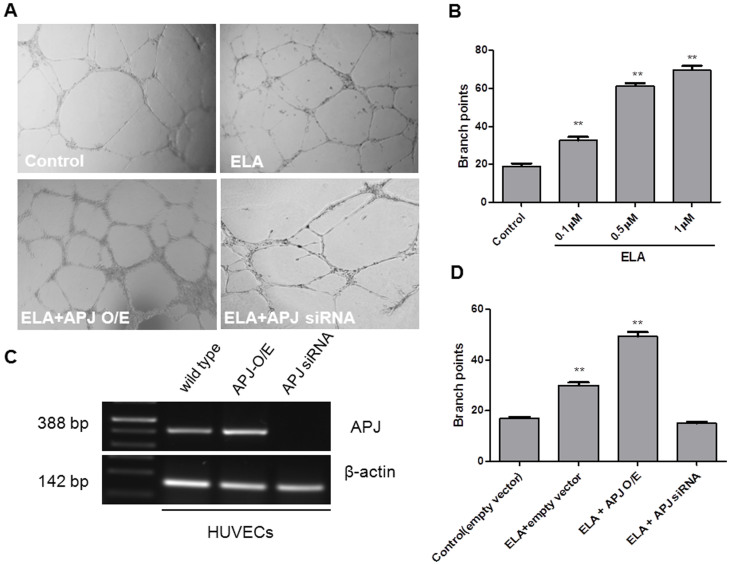
Stimulation of tubule-like structure by ELA-APJ signaling. (A), Representative images illustrating tubule-like formation in HUVECs in the absence or presence of ELA (0.5 μΜ), and in APJ-overexpressing or knocking-down HUVECs. (B), Reverse transcription PCR of human APJ expression of HUVECs infected with APJ or APJ siRNA lentiviral constructs. (C), Dose-dependent increase of the number of branch point by ELA in HUVECs. (D), APJ-dependent effect of ELA on tubule formation. HUVECs infected with empty vector (Control), APJ-overexpressing (APJ O/E) or APJ siRNA vectors were treated without or with ELA (0.1 μΜ). Values are mean + SEM (n = 5); **p < 0.01, compared to empty vector control without ELA.

ELA is a secreted hormone and expressed in adult kidney and prostate, and may, therefore, circulate in blood. Since ELA is required for normal development of cardiovascular system, blood vessel could be a target of ELA action. Vascular tube formation assay is a well-used *in vitro* study to assess angiogenic factors and conditions in which vascular cells^13^, [e.g. human umbilical vascular endothelial cells (HUVECs)], can form tube-like structures under pro-angiogenic conditions in culture wells coated with Geltrex containing extra-cellular matrix. The number of branch points is used as an indicator for the strength of angiogenic stimuli. As illustrated in Fig. 4, ELA treatment (0.5 μM) stimulated the formation of tubular structure (Fig. 4A). Quantitatively, the number of branch points was concentration-dependently increased by 71.9%, 222.8% and 266.7% in the presence of ELA at the concentrations of 0.1, 0.5 and 1 μM, respectively (Fig. 4B). To determine whether APJ mediated the angiogenic effect of ELA, we modulated APJ expression levels by over-expressing or knocking-down APJ (Fig. 4C) in HUVECs. In the cells over-expressing APJ, ELA (0.1 μM) increased the tubular formation by 190.2% and 63.3%, compared to empty vector control cells without and with ELA treatment, respectively. Conversely, ELA was not effective in inducing the formation of tubular structures in APJ-knock down cells (Fig. 4D). Together, the results indicate that ELA exerts angiogenic effect directly through activation of APJ.

### Relaxation of blood vessel by ELA

**Figure 5:**
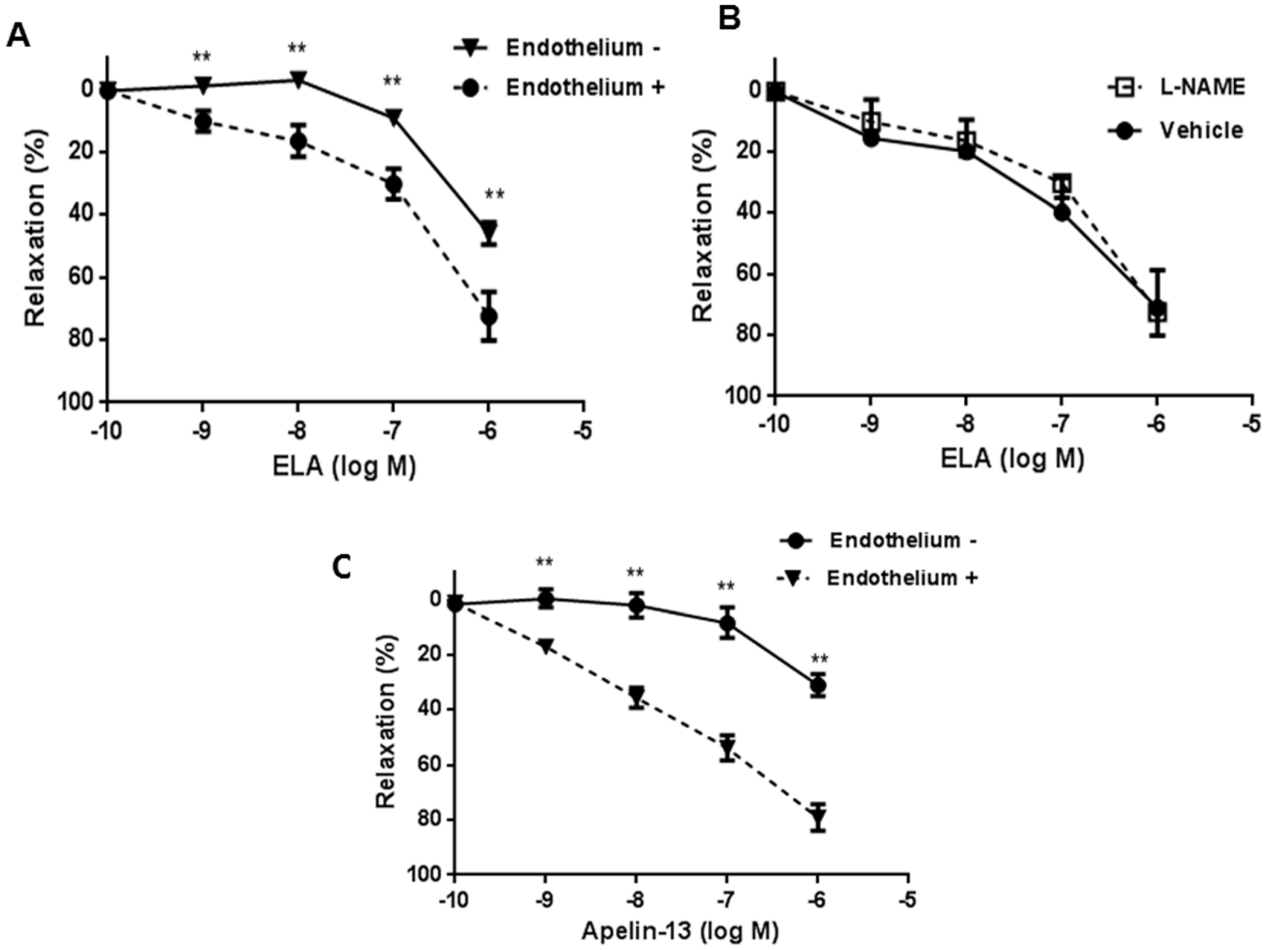
Relaxing effect of ELA and apelin-13 on blood vessels. Mouse aortic rings were first treated with vasoconstrictor U46619 (30 nM) to cause contraction and then with accumulated dose of ELA or apelin in intact or endothelium-depleted (denuded) vessels (A and C) for relaxation. Changes in tension were expressed as the percentage decrease in reference to precontraction tension. For L-NAME experiment, aortic rings were pretreated with 1 mM L-NAME for 30 min, then induced to contraction by U46619, followed by ELA. Values are means ± SEM. (n = 5–10). **p < 0.01, compared to aortas with endothelium at the same concentration points of the compound.

Apelin is reported to relax vascular tone^14^ and lower blood pressure. We next investigated whether ELA would regulate vascular tone using mouse endothelium-intact or endothelium-depleted (denuded) aortic rings. The aortic rings were induced to contraction with vasoconstrictor U46619 (final concentration, 30 nM). At the plateau of contraction, ELA or apelin-13 (10^−9^ to 10^−6^ M) was added to induce relaxation. As depicted in Fig. 5A, incubation with ELA relaxed the blood vessel in a concentration-dependent manner with maximum relaxation of 73.7% in endothelium-intact vessels. Notably, only about ~20% less relaxation was observed in denuded vessels at the corresponding concentrations, indicating an endothelium-independent relaxation by ELA. To further determine whether nitric oxide (NO) participated in the relaxation, the blood vessels were pretreated with L-NAME, an NO production inhibitor. No significant difference was observed between the L-NAME-treated and non-treated vessels, indicating that NO production is not required for ELA-mediated relaxation (Fig. 5B). The effect of apelin-13 on blood vessels was studied in parallel for comparison. Apelin-13 induced blood vessel relaxation in endothelium-intact vessels with maximum relaxation of 79% at 10^−6^ M, but the removal of endothelium blunted its relaxing effect greatly by 48% to 31% (Fig. 5C), indicating that apelin-13 appeared to induce vessel relaxation in a more endothelium-dependent manner than ELA did.

## Discussion

At the amino acid level, the matured ELA peptide shares 59% and APJ shares 54% identity between the zebrafish and human, suggesting a functional conservation between the two species. We show here that ELA is expressed in human adult embryonic stem cells and adult kidney, and activates human APJ in respect of its activities to suppress cAMP production, and to induce ERK1/2 phosphorylation and calcium mobilization. Functionally, ELA stimulates angiogenesis in human HUVECs and relaxes mouse aortic vessels. Thus, the ELA-APJ signaling is conserved not only at the molecular level but also in functionally in vertebrates.

Apelin was considered the only endogenous ligand for APJ since isolation of the apelin peptide. Apelin is expressed in multiple tissues including adipose tissue, heart, vasculature, brain and kidney, whereas APJ is more broadly expressed in tissues such as heart, blood vessels and adipose tissue. Decade of investigations suggest that apelinergic signaling is a multifaceted regulator of homeostasis^12^, especially for the cardiovascular system and water-electrolyte balance. At the receptor level, APJ is a GPCR coupled to Gi and/or Gq^15^,^16^. Activation of APJ results in biochemical responses including suppression of cAMP production, activation of ERK phosphorylation, stimulation of ENOS and mobilization of calcium, and in functional changes such as enhanced angiogenesis^17^,^18^, increased heart contractility^19^,^20^, and modulations of vascular tone^9^,^21^. Notably, infusion of apelin or apelin analogs appears to have cardioprotective effect in various animal models^22^,^23^,^24^,^25^,^26^,^27^, suggesting that APJ agonists could be a therapeutic reagent for heart failure.

The essential role of ELA in the development of heart and vasculature in zebrafish^10^,^11^ and conservation of ELA and APJ at the peptide level in vertebrates suggest a functional conservation in the species. To investigate the functionality of ELA and its relationship to APJ signaling in mammalian system, we examined the role of ELA in pathways known to be stimulated by the APJ receptor signaling. We first demonstrated that ELA is expressed in human embryonic (ESCs) and induced (iPSCs) pluripotent stem cells and adult kidney tissue, suggesting that ELA is a natural hormone in human system and may function during and after embryonic development. Next, we demonstrated that synthetic human ELA peptide causes internalization of human APJ, a typical response of GPCR to ligand, and that activation of APJ by ELA results in cAMP suppression, ERK activation and intracellular calcium mobilization in a dose-dependent manner, thus establishing that ELA acts on APJ in the human system. The pivotal developmental role of ELA in zebrafish suggests that the cardiovascular system is a main target of ELA action. Thus, we investigated whether ELA would modulate angiogenesis in HUVECs. Addition of ELA to HUVECs increased the tubular formation in a concentration-dependent manner. Importantly, overexpression of APJ increased tubular formation whereas knockdown abolished the ELA's action, establishing that APJ is required for the angiogenic effect of ELA. The effect of apelin on vascular tone is complex; a vasodilatory effect of apelin is reported^14^,^28^ but vasoconstriction is also noted in human saphenous vein^29^ and in vessels denuded of endothelium^28^. Thus, we examined the effect of ELA, in parallel with apelin-13, on vascular tone of mouse aortic rings. Vascular relaxation by apelin-13 was apparent in the intact blood vessel but was blunted greatly after denudation, indicating that endothelium mediated most of the relaxation effect, which is consistent with the findings observed in aortic ring of diabetic mice^14^. By contrast, ELA caused blood vessel relaxation in a less endothelium-dependent manner. Pretreatment of L-NAME did not abolish its relaxation effect, indicating that NO is not required for the effect. Thus, there appears a difference in the mechanism for blood vessel relaxation between ELA and apelin even though they act through the same receptor, APJ.

The discovery of ELA and its signaling through APJ has added a new functionality and regulatory mechanism to the apelinergic signaling system. APJ knockout (KO, APJ−/−) in mice results in more than half of early embryonic death with markedly deformed vasculature and heart^30^. The mice (APJ−/−) that had survived to birth displayed defects in their heart structure and reduction in myocardial contractility^30^,^31^. By contrast, the apelin knockout mice were born apparently healthy, a strong indication that other endogenous molecule(s) may likely signal via the APJ receptor^32^. It is, therefore, logical to reason that ELA would play an essential role for normal development of the cardiovascular system during embryonic development in mice as it does in zebrafish. In human adults, ELA transcripts are found only in the kidney and prostate^10^. Our RT-PCR analysis directly demonstrated ELA is selectively expressed in human kidney as well as in pluripotent stem cells. The predominant expression of ELA in kidney is in line with the essential role of ELA in cardiovascular system development in that the kidney, together with the heart, functions to maintain fluid balance and blood pressure. Thus, ELA may act as a paracrine or endocrine hormone regulating the circulation system, which deserves further investigation.

It is apparent that ELA and apelin act through the same receptor APJ. Whether the downstream signaling pathways and biological functions of the respective ligands are similar or not will be a subject of future studies. Our blood vessel relaxation studies have shown that ELA and apelin dilate the blood vessel by dissimilar mechanisms; ELA in a less-endothelium-dependent manner whereas apelin in a predominantly endothelium-dependent fashion. Indeed, the concept of biased agonism has been introduced to explain the distinctive cardiac responses to trans-aortic constriction (TAC) in mice^33^. In response to TAC, apelin-KO mice develop cardiac hypertrophy, whereas APJ-KO mice are resistant to the hypertrophy, suggesting that the TAC-induced hypertrophy is mediated by APJ, but not through apelin signaling. In addition, a significant decrease in water intake and inability to concentrate urine have been noted in APJ-KO mice^34^, but not reported in apelin-KO mice. These discrepancies in phenotype between the APJ-KO and apelin-KO mice suggest the presence of apelin-independent signaling pathway and a possible participation of ELA.

Recently, the therapeutic potential of apelin as a reagent for heart failure has been increasingly recognized. Several independent studies have shown that apelin and its analogs have cardiac-protective effect and can increase heart contractility, reduce infarction size and promote cardiac stem cell mobilization to the infarct area in the mouse heart failure model of coronary artery ligation^22^,^23^,^24^,^25^,^26^,^27^. Whether ELA has a similar or stronger cardiac protective function in mammals as deduced from its developmental role in cardiomyocytes formation in the zebrafish warrants further studies.

In summary, we here present data that the ELA-APJ signaling pathways initially discovered in zebrafish study are conserved and functional in vertebrates, which establishes a scientific basis for further functional studies to further understand the biological significance of these pathways in mammalians. From the perspective of clinical science, we expect that ELA and its analogs would help to derive functional cardiomyocytes at higher efficiency and may have therapeutic application in heart failure conditions.

## Methods

### Chemicals

The matured form of ELA peptide of 32-amino acid, QRPVNLTMRRKLRKHNCLQRRCMPLHSRVPFP, and apelin-13, QRPRLSHKGPMPF, were purchased from GenScript (Piscataway, NJ), at more than 98% purity. Other chemicals were from Sigma (St. Louis, MO) unless otherwise stated.

### Cell culture

Chinese hamster ovary (CHO) cells were from ATCC (Manassas, VA) and grown in Ham's F12 medium supplemented with 10% fetal bovine serum (FBS), 100 μg/mL streptomycin and 100 U/mL penicillin. Human umbilical vein endothelial cells (HUVECs) were purchased from Lonza (Walkersville, MD) and cultured in basal medium supplemented with 0.2% endothelial cell growth supplement (EnGS), 5 ng/mL recombinant human EGF, 50 μg/mL ascorbic acid, 10 mM L-Glutamine, 1 μg/mL hydrocortisone hemisuccinate, 0.75 U/mL heparin sulfate and 2% FBS. HUVECs between passage 3 and 5 were used for all experiments.

### Plasmid construction

The human APJ cDNA was amplified from human fat tissue by PCR with NotI/SalI restriction sites and cloned into a Gateway pENTRa vector (Invitrogen, Carlsbad, CA). EGFP- APJ fusion construct was made by assembling PCR amplified-APJ and EGFP fragments into pENTRa by Infusion cloning (Clontech, Mountain View, CA). To make the lentiviral destination vector pSMPUW-CMV-DEST, a fragment of CMV promoter–ccdB was cloned into the universal lentiviral vector pSMPUW (Cell BioLabs, San Diego, CA). Standard Gateway LR cloning protocol was used to generate pLenti-APJ or EGFP-tagged APJ by using LR Clonase II according to the manufacturer's instructions (Invitrogen). All lentiviral vector plasmids were amplified in STBL3 bacteria (Invitrogen). The cDNA inserts of all constructs were confirmed by restriction enzyme digestion and DNA sequence analysis.

### Production of lentivirus

HEK293T cells were cultured in Dulbecco's modified Eagle's medium (DMEM) containing 10% FBS, 100 ug/mL streptomycin and 100 U/mL penicillin. Cells were allowed to reach 90% confluence at which they were transfected in the presence of DMEM with 1.2 ug of transfer vector, 1.2 ug of pCD/NL-BH*DDD (Addgene plasmid 17531) and 0.2 ug of pVSVG (Cell Biolabs) for each well of a 6-well plate using LipoD293 (Signal). LipoD293/DNA complex-containing medium was removed and replaced with fresh medium 16 hr post transfection. The supernatant containing the viral particles was collected at 48 and 72 hr following initial transfection, combined and aliquoted for storage at −80°C. Lentiviruses used for all experiments had a minimum titer of 5 × 10^5^ IFU/mL.

### Stable CHO cell lines expressing APJ or EGFP-tagged APJ

CHO cells at about 100% confluence were plated onto 6-well plates and infected by exposure to lentiviral supernatant and polybrene (0.8 ug/mL, Millipore, Billerica, MA). The CHO cells were incubated for 16 hr, then viral supernatant was replaced with the CHO growth medium for 8 hr. The viral infection was conducted twice. Twenty four hours after the second infection, the cells were fed with the growth medium supplemented with 1 ug/mL puromycin and puromycin-resistant cells were expanded for further studies.

### Semi-quantitative RT-PCR

Tissue total RNAs were purchased from Clontech. Total RNAs from human embryonic stem cells and induced pluripotent stem cells^35^ were extracted using TRIzol reagent (Invitrogen) according to the manufacturer's instructions. cDNAs were synthesized using AMV Reverse Transcriptase kit (Promega, Madison, WI) from 1 ug of total RNA, and used for detection of gene expression. The primer sequences and expected fragment sizes were as follows: APJ 5′forward primer 5′-CTGGTGGTGACCTTTGCCCTG-3′ and reverse primer 5′-AAAGCTGGGTCTAGAGTCGACCTAGTCAACCACAAGGGTCTCCT-3′ (388 bp), ELA forward primer 5′-CTGAGGTTTGTCACTAGAATGTGAA-3′ and reverse primer 5′-TAAGCAATCACGCTGTTGGCATCA-3′ (360 bp), β-actin forward primer 5′-AGAAAATCTGGCACCACACC-3′ and reverse primer 5′-GGGGTGTTGAAGGTCTCAAA-3′ (142 bp).

### cAMP assay

Intracellular cAMP levels were measured using a time-resolved fluorescence resonance energy transfer (TR-FRET) assay with an HTRF cAMP kit (Cisbio), as described previously^36^. Briefly, CHO cells expressing APJ were seeded at 20 μl/well with 10,000 cells in white, tissue-culture-treated 384-well plates (Greiner BioOne). After overnight incubation at 37°C with 5% CO2, varying concentrations of ELA in assay buffer were added, followed by forskolin stimulation solution (final concentration of 20 μM) and Ro 20–1724 (100 μM). Following a 30 min incubation at room temperature, the assay plates were read in TR-FRET mode (excitation = 320 nm; emission = 615/665) using the EnVision plate reader (PerkinElmer). The amount of cAMP is reversely correlated with the ratio of 665/615 nm.

### ERK1/2 phosphorylation assay

Intracellular pERK1/2 levels were measured using TR-FRET assay by a HTRF Cellul'erk kit (Cisbio) according to the manufacturer's instructions and as reported before^36^. Briefly, following overnight incubation of APJ over-expressing or wide type CHO cells, the growth medium was replaced with 100 μl/well Opti-MEM medium (ATCC) and incubated for additional 4 hr. Various concentrations of ELA were added to assay plate and incubated for 20 min at 37°C with 5% CO2, then the medium was aspirated and the plates placed on ice for 5 min followed by the addition of cell lysis solution (Triton × 100). The plates were then incubated at room temperature with gently rocking (for mixing) for 15 min. An aliquot of 16 μl/well of cell lysate was transferred to a 384-well Greiner white half-well plate and 2 μl/well of the d^2^-dye-conjugated anti-ERK1/2 antibody was added. Following a 2 hour incubation in the dark, 2 μl/well of the europium cryptate-conjugated anti-ERK1/2 antibody was added. After 2 hr incubation at room temperature in the dark, the plates were measured using the EnVision plate reader in TR-FRET mode (excitation = 320 nm; emission = 615/665).

### Intracellular calcium assay

Intracellular calcium was measured using the nonwash calcium assay Fluo8 kit (AAT Bioquest) according to the manufacturer's instructions^36^. Briefly, CHO cells stably expressing APJ or empty vector were seeded as above and incubated overnight at 37°C with 5% CO2. Next day, growth media was aspirated and calcium dye added. Following incubation for 30 min at 37°C and 30 min at room temperature, ELA at various concentrations in assay buffer was added and assay plates incubated at room temperature for 10 min. Then the plates were placed into a fluorescence kinetic plate reader (μCell, Hamamatsu). The basal fluorescence intensity was recorded 10 times at 1 Hz for 10 s. The results were normalized to the average basal fluorescence intensity in ratio and the peak response was used for the result calculation.

### Western blot analysis

CHO cells were homogenized in lysis buffer containing 50 mM Tris-HCl (pH 6.8) and 2% SDS with freshly added protease/phosphatase inhibitor cocktail (Cell Signaling Technology, USA). The protein content was determined using a protein assay kit (Thermo Fisher Scientific, Waltham, MA). Each sample with equal amount of proteins was mixed with 5 × SDS samples buffer, boiled for 5 min, and separated on 10% sodium dodecyl sulphate-polyacrylamide electrophoresis (SDS-PAGE) before transferring the proteins onto a PVDF membrane. The membranes were then blocked at room temperature for 1 hr with 5% milk in Tris-buffered saline-Tween (TBST), followed by subsequent incubation at 4°C overnight in TBST containing the different primary antibodies (1:1000 dilution). After washing 3 times with TBST, the membranes were incubated for 2 hr in TBST containing the horseradish peroxidase-conjugated secondary antibodies (1:2000 dilution), followed by washing again with TBST for 3 times, then underwent detection by the chemiluminescence system. Extracellular signal-regulated kinase (ERK1/2, Cat.# 9102) and phosphor-ERK1/2 (Cat.# 9101) were purchased from from Cell Signaling Technology (Danvers, MA), and HRP-conjugated GAPDH monoclonal antibody (Cat.# HRP-6004) from ProteinTech Corporation (Chicago, IL).

### Internalization assay

HEK293 cells stably expressing EGFP-tagged APJ receptor were serum starved for 24 hr, and treated with ELA (500 nM), and analyzed by live fluorescence microscopy at time intervals following the treatment. For washout experiment, the cells were incubated with ELA for 1 hr and then replaced with serum-free basal medium, and images were taken 1 and 4 hr after the washout. Confocal imaging was performed with a Zeiss LSM510 microscope.

### *In vitro* angiogenesis (tubular formation) assay

Tubular formation assay for angiogenesis was conducted in 96-well plates which were coated with 50 μl of Geltrex (Life Technologies, A14133-02) and left for polymerization by incubation for 1 hr at 37°C. HUVECs (10,000/well, 3–5 passages) in basal medium (Lifeline, LM-0002) containing endothelial cell growth supplement (EnGS, Lifeline, LS-1018) with FGF (50 ng/mL) and/or different concentration of ELA (0.1, 0.5, 1 μM) were then plated on the Geltrex and incubated overnight at 37°C with 5% CO_2_. Quantification of the tubular formation was performed after 10 hr of culture by calculating the number of branching point formed in each well under an inverted phase contrast photomicroscope (Olympus IX50), as described^37^.

### Assays of endothelium-dependent and –independent vasorelaxation

Aortas were isolated from 8–10 weeks male wild-type C57BL/6 mice, cut into 3-mm rings and mounted in organ chambers (PowerLab, AD Instruments, CO, USA) in Kreb's buffer as described previously^38^,^39^. The contractile response was elicited by vasoconstrictor U46619 (30 nM) to produce contraction. At the plateau of contraction, accumulative ELA or apelin-13 (10^−9^ to 10^−6^ M) were added into the organ bath to induce relaxation. In some preparations, the endothelium was mechanically removed from denuded arteries by gentle rubbing off the intimal surface with a wooden stick. To determine whether nitric oxide participates in relaxation, aortas were pretreated with 1 mM L-ng-nitroarginine methyl ester (L-NAME) for 30 min, then induced to contraction by U46619, followed by ELA as described above. The effectiveness of endothelium removal and NOS inhibition were confirmed later by an inability of acetylcholine to relax the arteries.

### Statistical analysis

Data are expressed as means ± the standard error of the mean (SEM). Statistical analyses were performed by using of two-way ANOVA or Student's *t*-test. *P* < 0.05 was considered statistically significant.

## Author Contributions

Z.W., D.Y., M.W., Q.W., J.K., R.Y., K.Q., W.W. and C.S. performed the experiments. Z.W., D.Y., M.W., Q.W., J.K. and D.G. prepared the figures. Z.W., D.Y., Q.W., A.S., M.Z., W.Z. and D.G. participated in design of the experiments. All authors contributed to the writing of the manuscript.
